# Initiative of clinical single‐cell biomedicine in clinical and translational medicine

**DOI:** 10.1002/ctm2.1173

**Published:** 2023-01-11

**Authors:** Fangming Liu, Xuanqi Liu, Charles A Powell, Chengshui Chen, Yiming Zeng, Hao Fang, Xiangdong Wang

**Affiliations:** ^1^ Department of Pulmonary and Critical Care Medicine Zhongshan Hospital Shanghai Institute of Clinical Bioinformatics Shanghai Engineering Research for AI Technology for Cardiopulmonary Diseases Fudan University Shanghai Medical College Shanghai China; ^2^ Division of Pulmonary Critical Care and Sleep Medicine Icahn School of Medicine at Mount Sinai New York City New York USA; ^3^ Quzhou People's Hospital The Quzhou Affiliated Hospital of Wenzhou Medical University Quzhou China; ^4^ Center of Molecular Diagnosis and Therapy The Second Hospital of Fujian Medical University Quanzhou China; ^5^ Department of Anesthesiology Zhongshan Hospital Fudan University Shanghai China

**Keywords:** clinical, disease, medicine, single‐cell, translational

## Abstract

With rapid developments of single‐cell sequencing and multi/trans‐omics, clinical single‐cell biomedicine is a new and emergent discipline to integrate single‐cell molecular and clinical phenomes and uncover new disease‐specific diagnoses and therapy. The journal of Clinical and Translational Medicine (CTM) launches the first CTM initiative of clinical single‐cell biomedicine (cscBioMed) to promote the discovery and development of single‐cell‐based biology and medicine, speed the translation from single‐cell biology into clinical application, and improve early diagnosis and therapy for human diseases. The cscBioMed initiative is speeding translational processes from circulating single‐cell RNA sequencing into routine measures in clinical biochemistry of haematology, from spatial transcriptomics into single‐cell pathology, and from single‐cell‐based biomarkers and targets into clinical diagnostics and target drugs. With a clear goal, we expect that cscBioMed will benefit human health by establishing a clinical single‐cell dynamic monitoring and early predicting system and by improving diagnosis and treatment.

Single‐cell‐based research has been developing for decades and has resulted in a new discipline of single‐cell biology.^1^, ^2^, ^3^ With the rapid development of single‐cell technologies, single‐cell biology science has shown an impact on the identification and development of disease‐specific biomarkers. The deep understanding of single‐cell molecular and morphological phenomes provides new insights into the pathogenesis of human diseases and unites biological and medical science in the discipline of single‐cell biomedicine.^4^, ^5^, ^6^ Single‐cell systems biomedicine was further advanced by multi‐dimensional and molecular explorations of single‐cell nuclear elements, signal networks, inter‐organelle communication, and metabolism using single‐cell multi‐omics and trans‐omics.^7^, ^8^, ^9^, ^10^ More recently, human single‐cell atlases have had a great impact on disease diagnosis and therapy, by discovering new cell subsets, disease‐specific biomarkers, and therapeutic targets, monitoring dynamic responses to therapy and predicting prognosis.^11^ However, there is an urgent need to clarify the specificities of altered human single‐cell atlases in clinical phenomes, disease nature, severity, duration, stage, and response to therapy.

The journal of Clinical and Translational Medicine (CTM) launches the first CTM initiative of clinical single‐cell biomedicine (cscBioMed) to promote the discovery and development of single‐cell‐based biology and medicine, speed the translation from single‐cell biology into clinical application, and improve early diagnosis and therapy for human diseases. The cscBioMed has a clear goal to more accurately ensure the quality of human health and life using single‐cell technologies. In order to achieve the goal , we organize the resources of single‐cell biomedicine and create the new initiative of cscBioMed. The initiative is targeting acute and chronic infections and inflammations, cancers, and explosive epidemic diseases as monitoring and therapeutic areas, bus also at evaluation and prediction of health, sub‐health, and ageing. This is an international call for preclinical and clinical scientists and physicians to present the perspective hypotheses, creative thoughts, and advanced investigations for the discovery and development of cscBioMed. The important missions for cscBioMed are to better understand associations between single‐cell molecular and clinical phenomes, interactions among genes, proteins, and transcriptional factors, morphologies of spatial organization and remodelling, identities of cell subpopulations, and assemblies of an artificial intelligent cell.

The cscBioMed initiative is speeding translational processes from circulating single‐cell RNA sequencing (scRNA‐seq) into routine measures in the clinical biochemistry of haematology. There is rapidly growing evidence that circulating single cells including leukocytes, red blood cells, and platelets are measured in physiological and pathological conditions. Circulating scRNA‐seq identities of new cell subsets or biology‐ and disease‐specific clusters/subsets demonstrate the comprehensive capacity of the systemic immune response during the progression of the disease. Circulating scRNA‐seq can be a new measure of clinical biochemistry to monitor dynamical alternations of immune function and hematological hemostasis, although there remain a large number of challenges to be overcome.^12^ In addition to the challenge of confirming the accurate cell subset identities, other factors (e.g., hydrodynamics, movements between organs, transit between functional states, and short half‐life) may also influence the physiological reference ranges of each subset, impact the biological and pathological significance of subset number and percentage, and contribute to disease‐specific alterations. To meet the clinical standard of biochemistry, the settlement of the subset reference range will require a large population of healthy participants in each age group, gender, and race categories. For example, scRNA‐seq data from 1.27 million peripheral blood mononuclear cells from 982 donors demonstrated that 66% were only identified as cell state‐specific effects from the dynamic analysis of expression quantitative trait locus.^13^ In addition to blood samples, cscBioMed will pay special attention to single‐cell phenomes and subsets in other biopsies, for example, urine, cerebrospinal fluid, and sputum, although studies on those aspects remain limited. For example, cell types, subsets, and interactions in human nasal washing fluid measured by scRNA‐Seq were found to represent host and viral transcriptional profiles and epithelial/immune cell responses at infective spatialization to coronavirus disease 2019 and influenza.^21^

The cscBioMed initiative promotes the development of single‐cell pathology for the detection of single‐cell morphological phenomes, identities, and spatialization. Tissue morphological characteristics can be re‐constructed by labelled tissue single cells with corresponding transcriptomic identities and profiles from scRNA‐seq, although the accuracy of cell spatial specificities and variabilities remains to be improved, especially in the brain that is characterized by rich and complex intercellular connections and communication.^14^ Studies on human tissue scRNA‐seq demonstrate immune cell clusters and interactions in the tumour microenvironment and define new identities of cancer cells and new categories of cancer subtypes. As part of spatiotemporal molecular image and medicine, tissue scRNA‐seq paired with spatial transcriptomics can provide comprehensive information on histological stratification, pathological phenomes, and molecular changes, even though the approaches still require optimization and standardization.^15^, ^16^, ^17^ The initiative will extend the concept of ‘single‐cell pathology’ into ‘single‐cell image’, including images from computed tomography, nuclear magnetic resonance, and ultrasound. Clinical images can be enriched with histological stratifications, cell types and subsets, and transcriptomic profiles by integrating image, pathology, scRNAseq, and spatial transcriptome.^16^, ^17^ The combination of image mass cytometry with defined target biomarkers can demonstrate pathological spatial phenomes, distributions, heterogeneity, and stratification of tumour and stromal single cells to characterize intercellular connections and spatial patterns in the microenvironment and uncover new subtypes of cancer and correlations with clinical outcomes.^18^ This particular method is suitable for validation of the biomarker panels discovered from scRNA‐seq integrated with spatial transcriptomics or multi‐/trans‐omics, for translating comprehensive and spatiotemporal single‐cell phenomes into pathological measures and for developing clinical diagnostics of single‐cell pathology in large‐scale clinical applications. Single‐cell pathology may provide direct information to identify new histological phenomes, subtypes, and categories of disease, severity, and response to drugs. Integrating single‐cell multi‐omics with pathological spatialization and temporalization makes it possible to create the artificial intelligence‐based ‘whole cell model’^19^ from single‐cell biology into clinical values.

The cscBioMed initiative is accelerating the process from the discovery and development of disease‐specific diagnostic biomarkers and therapeutic targets on the basis of single‐cell multi‐omics/trans‐omics into clinical diagnostics and target drugs. Different from bulk multi/trans‐omics, single‐cell multi/trans‐omics provides multi‐dimensional and comprehensive insights for locating more precise cell types of target molecules, defining roles in intercellular functions, and clarifying specificities of intercellular and interorganellar target‐dominated signals. More recent studies demonstrated the value and impact of single‐cell multi‐omics in detecting various alternations of microenvironmental cardiac cell status, spatialization, and interactions with other cells, and highlighted the multi‐layer molecular networks and cell type‐ or phenome‐based remodelling in cardiac disease.^20^ Different from scRNA‐seq, single‐cell multi/trans‐omics presents a stereoscopic regulatory network and functional view of target‐oriented molecular panels, illustrates intercellular heterogeneities and interactions, and uncovers the relationship between target spatialization and temporalization, disease nature and phenomes, and patient outcomes. The Initiative will explore the special impact of single‐cell transcriptomic profiles in DNA mutations, modifications, and regulations, in target‐based individualized therapy, and in immunotherapy. Thus, the validation of scRNA‐seq‐based discoveries is a critically important process in translational medicine and science, with current emphasis on defining the specificity of single‐cell molecular phenomes and functions and correlating with clinical phenomes and response to therapies.

The cscBioMed initiative is gathering international single‐cell experts and working to translate the discovery and development of clinical challenges‐oriented and based single‐cell knowledge, diagnoses, and therapies, in partnership with scientific communities, universities, hospitals, and organizations. The Initiative strives to better understand and solve practical challenges during implementation, for example, how to make the expensive testing costs acceptable, complex measures applicable, required equipment certifiable, comprehensive processes standardizable, massive‐scale data analyses efficient, clinical explanations accurate and reliable, quality controls accessible, and clinical physicians understandable. It is also necessary to have specific regulations and protocols to apply scRNA‐seq in clinical practice. To meet those challenges, the cscBioMed initiative will accelerate the translational process to ensure that the measures of clinical scRNA‐seq and single‐cell pathology are accurate, efficient, repeatable, inexpensive, user‐friendly, and accessible. This will provide new opportunities to identify new cell types and subsets, generate a comprehensive information on immune function evaluation, define disease‐ or therapy‐specific heterogeneity and cell‐cell interaction, and develop biomarkers and targets specific to disease nature, severity, and outcome. We expect that cscBioMed will benefit human health by establishing a clinical single‐cell dynamic monitoring and early predicting system and by improving diagnosis and treatment.

## CONFLICT OF INTEREST

The authors declare that they have no conflict of interest.

## FUNDING INFORMATION

Not applicable.
